# The complete mitochondrial genome of *Camellia nitidissima* (Theaceae)

**DOI:** 10.1080/23802359.2023.2209211

**Published:** 2023-05-15

**Authors:** Hexia Liu, Liu Qin, Yuling Chen, Saiying Xu, Xingwen Zhou, Yulin Zhu, Bo Li

**Affiliations:** a College of Biology and Pharmacy, Yulin Normal University, Yulin, China; b Key Laboratory for Conservation and Utilization of subtropical Bio-Resources, Yulin Normal University, Yulin, China; c College of Architecture and Planning, Fujian University of Technology, Fuzhou, China

**Keywords:** Mitochondrial genome, medicinal plant, *Camellia nitidissima*, phylogenetic analysis

## Abstract

The mitochondrial genome of *Camellia nitidissima* was sequenced by Illumina and Pacbio sequencing. The results of sequences showed that a total length was 949,915 bp, and the GC content was 45.7% in assembled mitochondrial genome of *C. nitidissima.* 71 unigenes had been found, including 36 coding proteins and 35 non-coding proteins. Subsequently, the phylogenetic tree was built on 24 plants with the maximum-likelihood method, which had high bootstrap value and fited to the angiosperm phylogeny group classification (APG IV). The study’s findings unravel the taxonomic status of *C. nitidissima* and benefit the evolution study.

## Introduction

*Camellia nitidissima* C.W.Chi (1948), with golden flowers, belongs to Camellia genus of the family Theaceae, and it is mainly distributed in Guangxi, China. *Camellia nitidissima* is a kind of Chinese herbal medicine, whose flowers and leaves are particularly rich in saponins, flavonoids and polysaccharides (Hou et al. 2018), and *C. nitidissima* can be used in treatment of dysentery, hypertension, pharyngitis and hematochezia (Wang et al. 2016; He et al. 2018). Recently, the studies of *C. nitidissima* mainly focus on the formation of flower color (Zhou et al. 2017; Li et al. 2019), the types of secondary compounds (Jiang et al. 2020), and the extraction of chemical substances (Lin et al. 2013), but the mitochondrial genome of *C. nitidissima* has not been reported. However, the mitochondrial genome of *C. nitidissima* benefits to clarify its evolutionary position, so we propose to sequence and assemble the complete mitochondrial genome of *C. nitidissima*, and provide valuable genomic information for phylogeny.

## Materials

**Figure 1.**
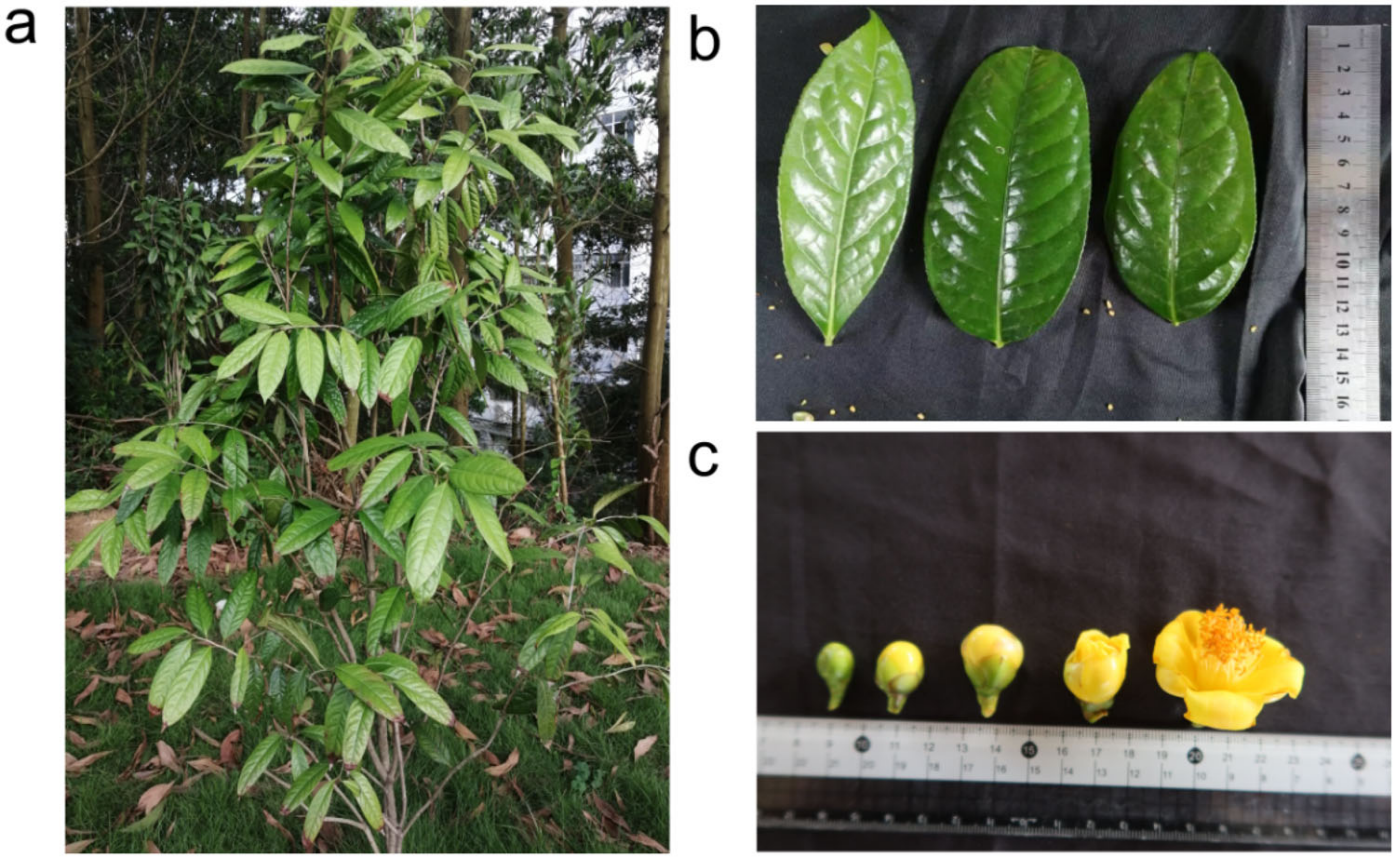
Morphology features of *C. nitidissima*. (a) Individual of *C. nitidissima*; (b) Mature leaves; (c) Buds and flowers. The photos of *C. nitidissima* were taken at the nursery of Yulin normal university, Yulin, Guangxi, China.

*Camellia nitidissima* is a shade-tolerant species and prefers warm, humid climate and acid soil with good drainage, and it is mainly propagated by seeds and cuttings. *Camellia nitidissima* were cultivated in the nursery of Yulin Normal University (N 22°40′58″, E 110°11′27″), Guangxi, China (Figure 1). The purple leaves of 7-year-old *C. nitidissima* plants were collected, cleaned, immediately frozen in liquid nitrogen, and stored at −80 °C. The studied specimen and genomic DNA of *C. nitidissima* were stored in the Herbarium of Yulin Normal University (https://syy.ylu.cn/index.html, Yulin Zhu, gxzyl@163.com) under the voucher number YLU20210011.

## Methods

Mitochondria of *C. nitidissima* was isolated from purple and young leaves with the density gradient centrifugation, and the contamination of genomic DNA was eliminated by DNase I (Promega, Madison, USA). DNA quality of mitochondria was checked by Qubit fluorometer (Thermo, Massachusetts, USA) and agarose gel electrophoresis. The sequencing library for Illumina was constructed using the NEBNext^®^ Ultra™ DNA Library Prep Kit (New England Biolabs, Suffolk, England) and sequenced by Illumina NovaSeq 6000 (Illumina, San Diego, USA). The SMRTbell libraries for Pacbio was constructed using the Express Template Prep Kit 2.0 (Pacific Biosciences, California, USA) according to the manufacturer’s protocol and sequenced by Pacbio Sequel II (Pacific Biosciences, California, USA). All of the above were entrusted to Biozeron company (Biozeron, Shanghai, China).

We used two strategies to assemble the mitogenome of *C. nitidissima*. In the first strategy, the short clean reads were de novo assembled with GetOrganelle v1.6.4 (parameters: -k 21, 65, 105) (Jin et al. 2020). Then, in order to extract the potential mitochondrial contigs, the assembled mitochondrial protein-coding genes were alignment with the plant mitogenome database by BLAST. Subsequently, the Pacbio long mitochondrial reads, which were used as bait, were mapped to the potential mitochondrial contigs by BLASR v5.1 with default parametersand assembled by Canu v2.1.1 (parameters: genomeSize = 140m, rawErrorRate = 0.3, correctedErrorRate = 0.045, corOutCoverage = 30) (Koren et al. 2017). In the second strategy, firstly, all Pacbio long reads were de novo assembled to get the draft contigs with Canu v2.1.1. Secondly, the short clean reads were mapped to the draft contigs by using BWA (parameters: bwa mem -t 4), and the draft contigs were improved by Pilon v1.22 (parameters: –fix all). Then, MUMmer 3.23 was used to check whether these contigs were circular. And finally, two corrected contigs, which were obtained from two assembly strategies, were align to each other by using MUMmer (parameters: nucmer –prefix). If these two contigs were identical, a master circle of the *C. nitidissima* mitogenome was correctly assembled. Mitochondrial genes of *C. nitidissima* were annotated by the GeSeq tool (Tillich et al. 2017), with the default parameters, to predict coding proteins, tRNA, and rRNA. And then those genes of functional annotations were performed to blast against (evalue < 1e-10) the non-redundant protein database (Nr), Swiss-Prot, Clusters of Orthologous Groups (COGs), and Kyoto Encyclopedia of Genes and Genomes (KEGG) and Gene Ontology terms (GO). Subsequently the position of each coding gene was determined using BLAST searches against ref mitochondrion genes in NCBI. The mitochondrial genome map was drawn by the OGDRAW tool. Based on 23 conserved protein-coding genes in the mitochondrial genomes, phylogenetic tree of *C. nitidissima* and 23 plants was constructed with maximum-likelihood estimation by PhyloSuite software with the best-fit model (GTR + F+R2) and 1000 bootstrap replicates (Zhang et al. 2020).

## Results

**Figure 2.**
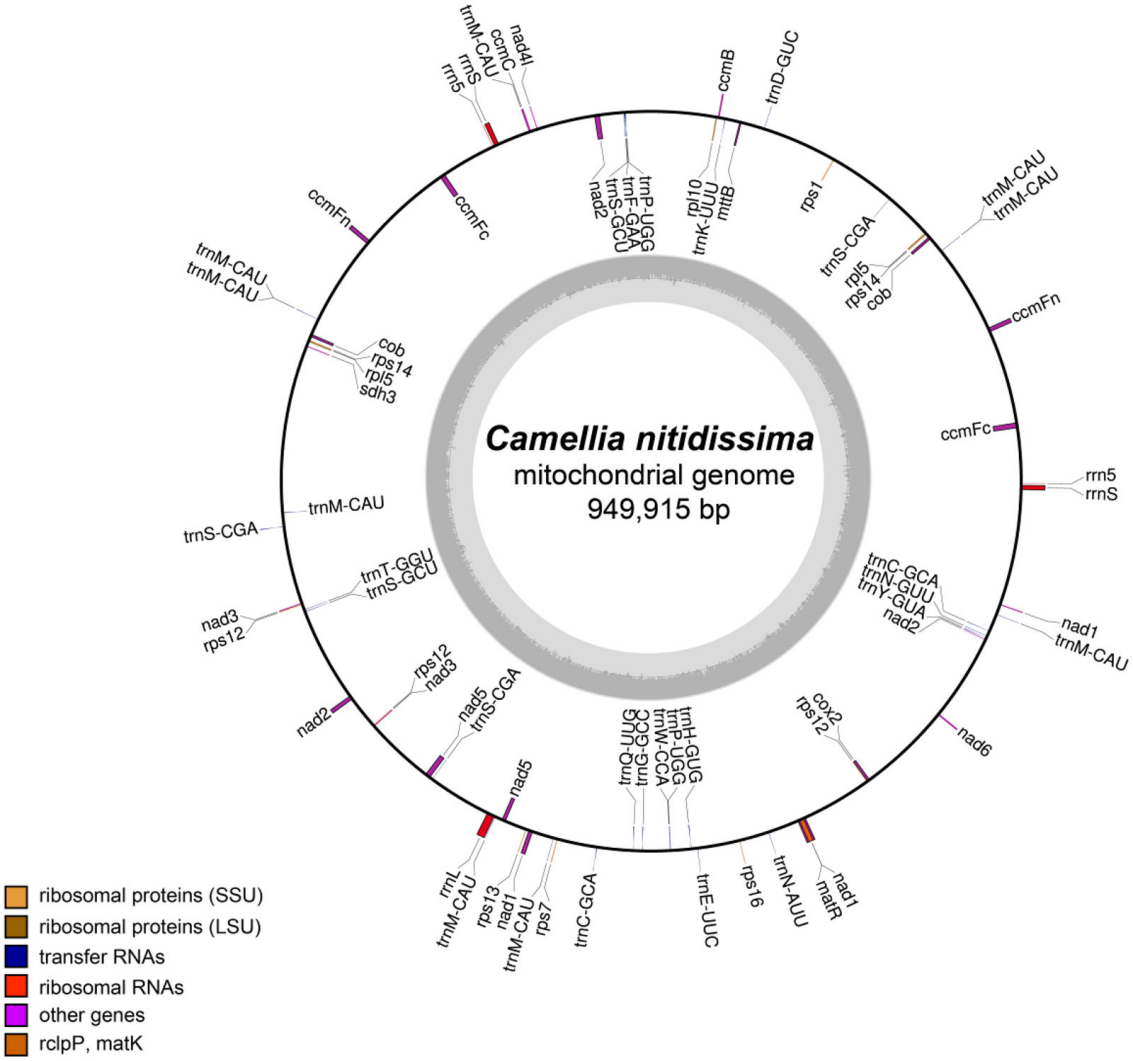
The gene map of the complete mitochondrial genome of *C. nitidissima*. Genes indicated in the inner circle are transcribed clockwise, and those indicated in the outer circle are transcribed counterclockwise. Different functional groups of genes are color coded. The darker gray corresponds to DNA G + C content, while the lighter gray corresponds to A + T content.

**Figure 3.**
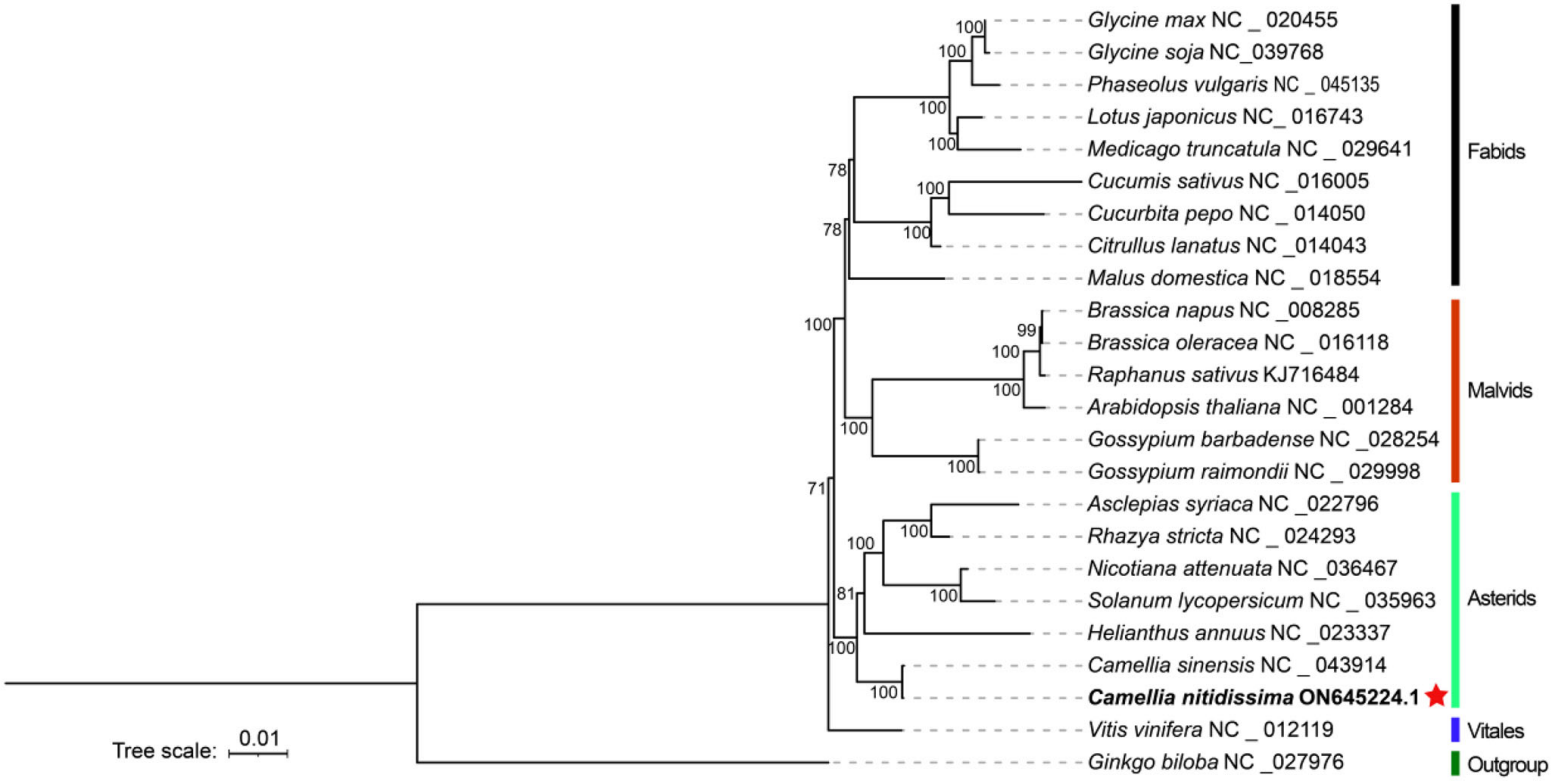
Used the *Ginkgo biloba* (NC_027976) as outgroup, the phylogenetic tree of 24 plants was constructed with maximum-likelihood method by PhyloSuite. The numbers above the branches were the bootstrap value. The 23 conserved protein-coding genes (*atp1*, *atp4*, *atp6*, *atp8*, *atp9*, *ccmB*, *ccmC*, *cox1*, *cox3*, *cytb*, *matR*, *mttB*, *nad1*, *nad2*, *nad3*, *nad4*, *nad5*, *nad6*, *nad7*, *rpl2*, *rps10*, *rps19*, *rps3*) in the mitochondrial genomes were used to construct the phylogenetic.

The sequenced results showed the 94.1 million reads and 14.1 Gb raw data were obtained by two sequencing technologies. The assembled mitochondrial genome of *C. nitidissima* was a single circle with a total length of 949,915 bp, and the GC content was 45.7%. In total, mean sequencing depth was 1044.91 ×(Figure S1, Table S1). There has been found 36 coding proteins and 35 non-coding proteins, including 30 transfer RNA (tRNA), 5 ribosomal RNA (rRNA), in the mitochondrial genome of *C. nitidissima*. The total length of coding proteins and non-coding proteins were 30,051 and 5,687 bp, accounting for 3.16 and 0.599% of the total genome length, respectively. 36 protein-coding proteins contained electron chain complex of oxidative phosphorylation (16), cytochrome c biosynthesis (6), ribosomal proteins (12), maturases (1) and transport membrane protein (1). In addition, 9 genes, such as *ccmFc*, *ccmFn*, *cob*, *nad3*, *rpl5*, *rps12*, *rps14*, *rrnS* and *rrn5*, have multiple copies in the mitochondrial genome of *C. nitidissima* (Figure 2). Five intron-containing genes (*ccmFc*, *nad2*, *nad4*, *nad1*, *nad5*) were found, which contained 15 introns totally. Based on 23 conserved protein-coding genes (*atp1*, *atp4*, *atp6*, *atp8*, *atp9*, *ccmB*, *ccmC*, *cox1*, *cox3*, *cytb*, *matR*, *mttB*, *nad1*, *nad2*, *nad3*, *nad4*, *nad5*, *nad6*, *nad7*, *rpl2*, *rps10*, *rps19*, *rps3*) in the mitochondrial genomes, the phylogenetic analysis showed that 24 plants were divided into five branches. The following sequences with GenBank accession were used: *Glycine max* NC_020455 (Chang et al. 2013), *Glycine soja* NC_039768 (Asaf et al. 2018), *Phaseolus vulgaris* NC_045135 (Bi et al. 2020), *Lotus japonicas* NC_016743 (Kazakoff et al. 2012), *Medicago truncatula* NC_029641 (Bi et al. 2016), *Cucumis sativus* NC_016005, *Cucurbita pepo* NC_014050 (Alverson et al. 2010), *Citrullus lanatus* NC_014043 (Alverson et al. 2010), *Malus domestica* NC_018554 (Goremykin et al. 2012), *Brassica napus* NC_008285 (Handa 2003), *Brassica oleracea* NC_016118 (Chang et al. 2011), *Raphanus sativus* KJ716484 (Jeong et al. 2016), *Arabidopsis thaliana* NC_001284 (Giegé and Brennicke 1999), *Gossypium barbadense* NC_028254 (Tang et al. 2015), *Gossypium raimondii* NC_029998 (Bi et al. 2016), *Asclepias syriaca* NC_022796 (Straub et al. 2013), *Rhazya stricta* NC_024293 (Park et al. 2014), *Nicotiana attenuate* NC_036467, *Solanum lycopersicum* NC_035963 (Xu et al. 2017), *Helianthus annuus* NC_023337 (Bock et al. 2014), *Camellia sinensis* NC_043914 (Rawal et al. 2020), *Camellia nitidissima* ON645224 (unpublished), *Vitis vinifera* NC_012119 (Goremykin et al. 2009), *Ginkgo biloba* NC_027976 (Guo et al. 2016). The *Ginkgo biloba* (NC_027976) used as outgroup was divided into one branch; the grape was divided into one branch alone; *C. nitidissima* and six plants were divided into one branch; two plants of Malvaceae and four plants of Cruciferous were divided into another branch and the remaining nine plants were divided into final branch (Figure 3). In the phylogenetic tree, *C. nitidissima* was clustered into a branch with the plants of Theaceae, Apocynaceae, Solanaceae and Compositae, which had high bootstrap value and fited to the angiosperm phylogeny group classification (APG IV). Moreover, *C. nitidissima* was closely related to *Camellia sinensis*, which all belonged to Camellia genus of the family Theaceae, with 100 bootstrap values. Therefore, it is thought that the phylogenetic analysis in this study can distinguish species of plant by using sequences of mitochondrial gene.

## Discussion and conclusion

The event of horizontal gene transfer can be identified by comparing mitochondrial genome of *C. nitidissima* with the chloroplast genome in future studies, which will provide a basis for the functional study of horizontal gene transfer sequences. This complete mitochondrial genome of *C. nitidissima* will benefit the evolution study, germplasm identification and development of molecular markers.

## Supplementary Material

Supplemental Material

## Data Availability

The genome sequence data that support the findings of this study are openly available in GenBank of NCBI at [https://www.ncbi.nlm.nih.gov] under the accession no. ON645224.1. The associated BioProject, SRA, and Bio-Sample numbers are PRJNA899601, SRR22240446, SRR22240447, and SAMN31665678, respectively.
