# Supplementary material for: The complete mitochondrial genome of Camellia nitidissima (Theaceae)
Source: Mitochondrial DNA B Resour. 2023 May 15;8(5):565–9. doi: 10.1080/23802359.2023.2209211 (PMC10187084; doi:10.1080/23802359.2023.2209211)
Supplement: Supplemental Material [file TMDN_A_2209211_SM3118.docx]

Table S1. Statistical analysis of coverage of sequencing in *C．nitidissima* mitochondrial genome

| Name of mitochondrial genome | Length | Covered Base | Total Depth | Coverage% | Mean Depth |
| --- | --- | --- | --- | --- | --- |
| *C．nitidissima* | 949,915 | 949,876 | 992,570,985 | 99.99 | 1044.91 |


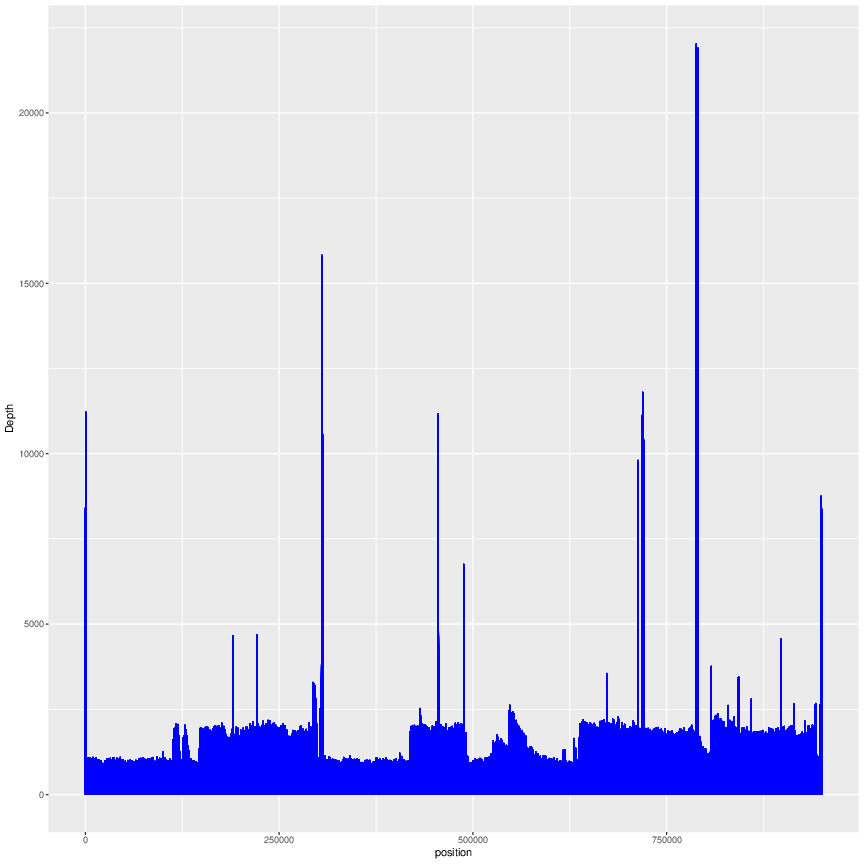


Figure S1. Coverage with sequencing depth of *C. nitidissima* mitochondrial genome. X and Y axis present nucleotide position of *C. nitidissima* mitochondrial genome and read mapping depth, respectively. The average mapping depths are 1044 ×.
